# Immunohistochemical and Molecular Genetic Analysis of Canine Digital Mast Cell Tumours

**DOI:** 10.3390/ani13101694

**Published:** 2023-05-19

**Authors:** David Conrad, Alexandra Kehl, Tobias Müller, Robert Klopfleisch, Heike Aupperle-Lellbach

**Affiliations:** 1 Department of Pathology, Laboklin GmbH & Co. KG, 97688 Bad Kissingen, Germany; aupperle@laboklin.com; 2 Department of Comparative Experimental Pathology, School of Medicine, Technical University of Munich, 81675 München, Germany; kehl@laboklin.com; 3 Department of Molecular Biology, Laboklin GmbH & Co. KG, 97688 Bad Kissingen, Germany; 4 Department of Bioinformatics, University of Würzburg, 97074 Würzburg, Germany; tobias.mueller@uni-wuerzburg.de; 5 Institute of Veterinary Pathology, Freie Universität Berlin, 14163 Berlin, Germany; robert.klopfleisch@fu-berlin.de

**Keywords:** dog, digit, toe, CD117, Ki67, KIT, grading, *c-kit*, PCR, sequencing

## Abstract

**Simple Summary:**

In veterinary medicine, methods such as histological grading, immunohistochemistry and mutation analysis of the *c-kit* gene are tools to assess the prognosis and treatment of canine cutaneous mast cell tumours. These methods have not yet been applied and evaluated on a large scale for mast cell tumours of the dog’s toe, as these digital mast cell tumours are considered a subset of the cutaneous forms. Mast cell tumours can be more aggressive at certain sites. Therefore, the aim of this study was to apply these methods to 68 dogs with digital mast cell tumours. Even though only a few digital mast cell tumours were histologically poorly differentiated, more than half had immunohistochemical findings that could indicate an unfavourable prognosis. Mutations in the *c-kit* gene were found as well. Moreover, French Bulldogs—a breed that tends to develop benign variants at other sites on the skin—were more likely to have poorly differentiated tumours. Although outcome data were not available, the results of this study may contribute to a better understanding of canine mast cell tumours of the toe.

**Abstract:**

Grading, immunohistochemistry and *c-kit* mutation status are criteria for assessing the prognosis and therapeutic options of canine cutaneous mast cell tumours (MCTs). As a subset, canine digital MCTs have rarely been explored in this context. Therefore, in this retrospective study, 68 paraffin-embedded canine digital MCTs were analysed, and histological grading was assessed according to Patnaik and Kiupel. The immunohistochemical markers KIT and Ki67 were used, as well as polymerase chain reaction (PCR) for mutational screening in *c-kit* exons 8, 9, 11 and 14. Patnaik grading resulted in 22.1% grade I, 67.6% grade II and 10.3% grade III tumours. Some 86.8% of the digital MCTs were Kiupel low-grade. Aberrant KIT staining patterns II and III were found in 58.8%, and a count of more than 23 Ki67-positive cells in 52.3% of the cases. Both parameters were significantly associated with an internal tandem duplication (ITD) in *c-kit* exon 11 (12.7%). French Bulldogs, which tend to form well-differentiated cutaneous MCTs, had a higher proportion of digital high-grade MCTs and ITD in *c-kit* exon 11 compared with mongrels. Due to its retrospective nature, this study did not allow for an analysis of survival data. Nevertheless, it may contribute to the targeted characterisation of digital MCTs.

## 1. Introduction

Mast cell tumours (MCTs) represent up to 21% of all canine cutaneous tumours [1,2,3,4,5] and are generally characterised by very unpredictable biological behaviour [6]. For some dog breeds, predispositions have already been reported; Boxers [3,4,7,8], Golden and Labrador Retrievers [3,4], French Bulldogs [3,4,7] and American Staffordshire Terriers [4] are often affected, whereas no gender predisposition could be identified [4,5]. The average age of dogs affected by MCTs is nine years [5], but they have also been described in dogs younger than one year of age [9].

Histological grading remains one of the cornerstones in the prognostic analysis of canine MCTs. The three-tier system developed by Patnaik and colleagues [8], in which tumours are classified into grades I to III, is still widely used. Unfortunately, a large number of MCTs are classified as grade II and are difficult to assess in terms of their biological behaviour, which weakens the prognostic value of this system [10]. In addition, interobserver variation has been reported [11]. Kiupel and colleagues [10] have addressed these problems, and subsequently developed a two-tier grading system that classifies tumours as low- or high-grade. The latter system has proven to be more prognostically significant, and is often used in addition to the Patnaik system [12].

There are various immunohistochemical markers used in canine MCTs. In this context, a meta-analysis by Freytag et al. [13] identified Ki67 and KIT as having prognostic value. Ki67 is a protein originally discovered in human research, whose cellular expression is closely related to the cell cycle. It is not found in cells that are in the resting phase, making Ki67 a suitable marker for mitotically active cells [14] and also a prognostic factor for canine MCT [13]. The receptor tyrosine kinase KIT (CD117) is a membrane-bound receptor that interacts with its ligand stem cell factor (SCF), and is not only located on mast cells, but also on the surface of many other cell types such as haematopoietic stem cells, interstitial cells of Cajal, melanocytes and germ cells; it plays an important role in the survival and differentiation of these cells, as summarised by Esteban-Villarrubia et al. [15]. Mast cells can show aberrant immunohistochemical patterns of KIT expression, e.g., a perinuclear pattern (pattern II) and a diffuse intracytoplasmic pattern (pattern III) [16]. The two aberrant patterns are correlated with increased cell proliferation and a higher histological grade of canine MCT [17].

The proto-oncogene *c-kit* encodes for the receptor tyrosine kinase KIT [15]. Imatinib, masitinib and toceranib are examples of receptor tyrosine kinase inhibitors (RTKI) that can inhibit KIT on an intracellular level [18]. In some cases, they can achieve higher response rates if a *c-kit* mutation is present [19,20]. However, imatinib was developed for treating human chronic myelogenous leukaemia [18] and is not approved for cancer treatment in small animals. According to the European Medicines Agency (EMA), the use of masitinib (Masivet^®^) is indicated if a *c-kit* mutation has been proven (https://www.ema.europa.eu/en/medicines/veterinary/EPAR/masivet, accessed on 16 February 2023), which requires genetic testing for *c-kit* mutations before starting treatment. This restriction does not apply to toceranib (Palladia^®^), as according to the EMA marketing authorisation, the detection of a *c-kit* mutation is not mentioned (https://www.ema.europa.eu/en/medicines/veterinary/EPAR/palladia, accessed on 3 May 2023).

Mutations of the *c-kit* gene were found in a variety of canine cutaneous MCTs, with these variants most frequently occurring in exon 11, and less frequently in exons 8, 9 and 14 [21,22,23,24,25]. In a study including 221 cutaneous MCTs, internal tandem duplications (ITDs) in exon 8 were associated with longer total survival [26]. Mutations in exon 9 seem to be involved in the development of resistance to the RTKI masitinib in cutaneous MCT [27]. It is interesting to note that in one study, Nakano and colleagues [25] reported on a canine MCT carrying a secondary mutation in exon 14, which was thought to be responsible for imatinib resistance.

ITDs in exon 11 were found in up to 50% of canine cutaneous MCTs, and correlate with higher grading [28,29], higher cellular proliferation activity [30], higher risk of metastasis at the time of surgery [29], and decreased survival times [21,26].

Commonly reported sites of cutaneous MCTs include the limbs, head and trunk [31,32]. These sites have often been the subject of research, whereas MCTs of the digit have barely been studied separately. Yet, up to 78% of all neoplastic processes on the toe are malignant tumours, and 4.3 to 10.5% of these are MCTs [33,34,35]. It has been observed that MCTs at certain locations such as mucocutaneous junctions or the inguinal region have a poorer prognosis than MCTs at other body sites [5,6]. This could also be related to the fact that tumours at these sites are difficult to resect with clean margins [5].

The aim of this study was to characterise canine digital MCTs with respect to their histological grading, immunohistochemical KIT patterns and proliferation activity (Ki67), as well as their *c-kit* mutations.

## 2. Materials and Methods

In this retrospective study, 68 MCTs of the canine digit were used, which were submitted for routine diagnostics between 2014 and 2022 to Laboklin GmbH & Co. KG, Bad Kissingen, Germany. In all cases, the initial diagnosis “mast cell tumour of the digit” had been made by a trained veterinary pathologist.

The inclusion criteria were that the MCT was of dermal origin, localised to the toe, and a paraffin-embedded tumour block was available prior to decalcification, because of its further use for immunohistochemistry (IHC) and DNA isolation. In addition, all cases that contained tumours of subcutaneous origin were excluded from the study.

Age, gender, breed and further information, if available, were collected. For additional information, the veterinarians were contacted by telephone. The exact site of the digital mass and a short report of the clinical course were documented whenever possible. The size of the tumour was either documented before cutting or, if this was not clearly visible, the diameter of the tumour was measured on the digitised specimen.

As all samples were submitted for routine diagnostic purposes and, moreover, were no longer needed for diagnostics, it was not required that we submit an animal testing request or obtain an ethics committee’s approval. This approach is supported by the decision of the local government (RUF-55.2.2-2532-1-86-5).

### 2.1. Histopathology

Toes were trimmed routinely as previously described [36]. One non-decalcified tumour site from each toe was used for further IHC and genetic analysis of mutations in *c-kit* exons 8, 9, 11 and 14. Sections were stained with haematoxylin and eosin (HE) and histologically evaluated by a trained veterinary pathologist as part of routine diagnostic procedures, and re-evaluated by H.A-L. and D.C.

Histological grading was carried out according to the known schemes of Patnaik et al. [8] into grade I–III, as well as into low- and high-grade according to Kiupel et al. [10]. Evaluation of histological tumour margins and analyses that included cell counting, such as mitotic count/1 or 10 high-power fields (HPF), cells with three or more nuclei/10 HPF, cells with bizarre nuclei/10 HPF and karyomegaly, were performed on digitised slides. For this purpose, the HE-stained sections were scanned with an Aperio AT2 Scanner (Leica Biosystems, Deer Park, IL, USA), digitised, and evaluated using the Aperio ImageScope software (v12.4.3.5008; Leica Biosystems, Deer Park, IL, USA). The area of 10 HPF was defined as 2.37 mm^2^, as described by Meuten et al. [37], and digitally plotted within the area of highest mitotic activity. As there is no standard classification for the histological assessment of MCT margins [38], we recorded the distance between tumour cells and healthy tissue at the narrowest point, and defined the tumour margins similarly to the work of Karbe et al. [39] as follows: clean (>3 mm), narrow (<3 mm), and infiltrated by tumour cells (<1 mm).

Due to the retrospective nature of this study, there was a lack of corresponding lymph node tissue in the individual cases. It was also not possible to determine whether the individual dogs were affected by solitary or multiple mast cell tumours, or if metastases were present. Clinical staging according to WHO recommendations was therefore not feasible.

### 2.2. Immunohistochemistry

For immunohistochemistry, 2 µm-thin sections of each formalin-fixed and paraffin-embedded (FFPE) tissue were mounted on coated slides (SuperFrost^®^ Plus, Menzel Gläser, Thermo Scientific, Waltham, MA, USA). After drying overnight at 58 °C, these sections were subjected to immunohistochemistry for analysis of KIT expression pattern and Ki67 expression. Pre-treatment for antigen demasking was performed at 96 °C with EDTA buffer (HIER T-EDTA, pH 9.0, Zytomed #ZUC029-500, Zytomed System GmbH, Berlin, Germany) in a commercial steam heater for 25 min. Primary antibodies (Table 1) known to cross-react with canine tissue were diluted in antibody dilution (#ZUC025-100, Zytomed System GmbH, Berlin, Germany) and applied on cover plates (Shandon Coverplate™, #A73310024, Thermo Fisher Scientific, Cheshire, UK). Positive controls were canine cutaneous basal cells (KIT) and canine lymph node tissue (Ki67). The sections were incubated with the primary antibodies at room temperature for 60 min, and the primary antibody was replaced with buffer for the negative controls. This was followed by the application of an enhancement reagent for 20 min and the application of an HRP polymer for 30 min, both at room temperature. Both reagents are components of a commercially available detection system (ZytoChem Plus HRP Polymer Kit, #POLHRP-100, Zytomed System GmbH, Berlin, Germany). Finally, the slides were incubated at room temperature for 10 min with a DAB (3,3′-diaminobenzidine) chromogen (#K3468, Dako Denmark A/S, Glostrup, Denmark), and counterstaining was performed with Mayer’s haematoxylin.

The KIT staining pattern was divided into the following three classes: pattern I, membrane-associated, pattern II, focal to stippled cytoplasmic, and pattern III, diffusely cytoplasmic [17]. The two cytoplasmic patterns II and III are summarised hereafter as aberrant patterns, according to Freytag et al. [13].

Evaluation of the Ki67-positive cells was carried out as already described by Webster et al. [30]. However, the analysis was also performed on digitised preparations. According to our calculations, the 1 cm^2^ grid area used by Webster et al. at 400 × magnification corresponds to an area of 250 × 250 µm in the digital field. Of these areas, five were placed in sections of high proliferative activity, the Ki67-positive cells in each field were digitally labelled, and the mean value of the sum was calculated.

### 2.3. Screening for Mutations in Exons 8, 9, 11 and 14 of c-kit

From the paraffin-embedded samples, 2 µm-thin layers of tumour tissue were removed with a microtome and then processed directly for DNA extraction, first using a QIAamp^®^ DNA FFPE Tissue Kit (Qiagen, Hilden, Germany) and subsequently a Clean & Concentrator Kit (Zymo Research, Freiburg, Germany), according to the manufacturer’s instructions. The sequences of exons 8, 9, 11 and 14 of *c-kit* were analysed using PCR amplification followed by Sanger sequencing. Primers are listed in Table 2. Bidirectional Sanger sequencing of all PCR products was performed on an ABI 3130 Genetic Analyzer (Life Technologies, Carlsbad, CA, USA) using a BigDye Terminator v1.1 Cycle Sequencing Kit (Life Technologies, Carlsbad, CA, USA), according to standard protocols. Sequencing files were analysed using Sequence Scanner software (version 2; Applied Biosystems, Thermo Fisher Scientific, Waltham, MA, USA).

### 2.4. Statistical Analysis

Statistical significance analyses were evaluated using IBM SPSS Statistics (version 29, IBM, Armonk, NY, USA). Using the Kruskal–Wallis test, Patnaik grading results were compared with the following parameters: mitotic figures (for statistical purposes within 10 HPF), Ki67 count (more or less than 23 positive cells per grid area), and KIT pattern (physiological versus aberrant). The results of the Kiupel grading were compared with the same parameters using the Mann–Whitney-U (MWU) test. 

Likewise, the impact of ITD in *c-kit* exon 11 on Patnaik grading and mitotic figures/10 HPF was analysed using the MWU test. For comparing ITD in *c-kit* exon 11 and the remaining binary codable variables (Kiupel grading, Ki67 count and KIT pattern), Fisher’s exact test was applied.

The Ki67 and KIT pattern were compared to each other using the MWU test and a Benjamini–Hochberg multiple testing correction, as implemented in the statistical framework R version 4.2.2 [40].

Finally, Firth’s bias-reduced penalized-likelihood logistic regression was applied as implemented in the R package “logistf” [41] to compare the results of the Kiupel grading with the clinical data (gender, castration, tumour size and age), KIT pattern, Ki67 count and ITD in *c-kit* exon 11. To start with, the full model that includes all covariates is used. Then, the ideal model is selected by a backward model selection procedure based on a penalised likelihood ratio test, as implemented in the logistf R package. Since the Patnaik factor is an ordered categorical variable with three levels (grade I–III), a generalisation of the typical logistic regression had to be applied. An “ordered logistic regression” was used here as implemented in the “polr” function in the R package MASS [42]. As with the Kiupel grading, at first, the full model is used and then a backward model selection procedure is applied, this time based on the Bayesian information criterion (BIC).

## 3. Results

### 3.1. Case Description

The age of the 68 dogs that were included in this study ranged from 2 to 15 years, with a median age of 8 years. In one case, the age was unknown (Table S1). Some 35 animals were male (24 intact, 11 castrated), and 33 animals were female (21 intact, 12 spayed). The following breeds were represented: 13 mongrels, 7 French Bulldogs, 7 Labrador Retrievers, 6 Boxers, 6 Golden Retrievers, 5 Pugs, 2 Bernese Mountain Dogs, 2 German Shepherds, and 2 Yorkshire Terriers. There was only one animal of each of the following breeds: American Staffordshire Terrier, Beagle, Border Collie, Boston Terrier, Bracke, Briard, Bullmastiff, Chihuahua, Cocker Spaniel, Coton de Tuléar, Dogo Argentino, English Toy Terrier, Havanese, Jack Russell Terrier, Maltese, Munsterlander, Puli, and Siberian Husky.

MCT affected the forelimb (*n* = 35) more often than the hindlimb (*n* = 19). In 14 cases, this information was not available. Telephone enquiries were made to record the further course and the current health status of the animals. We also asked whether alternative or additional therapies (especially the use of receptor tyrosine kinase inhibitors) were carried out in addition to toe amputation, in order to be able to analyse the correlation between the presence of *c-kit* mutations and a successful or failed treatment. However, it turned out that this information was no longer available in the majority of cases. The reason for this was that due to the retrospective nature of this study, some of the cases dated back to 2014, and the relevant information was no longer available in the records of the clinicians contacted. In addition, the animals were often only presented to the clinicians for toe amputation, and were afterwards treated elsewhere. We therefore decided not to statistically investigate survival times and the influence of mutations in *c-kit* on therapeutic success, in order to avoid generating non-valid data.

### 3.2. Grading and Pathological Findings

**Figure 1:**
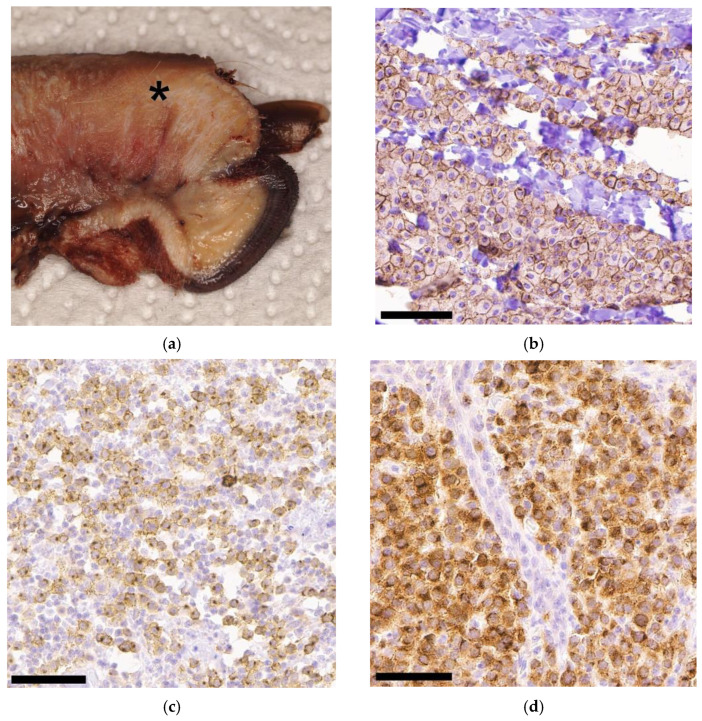

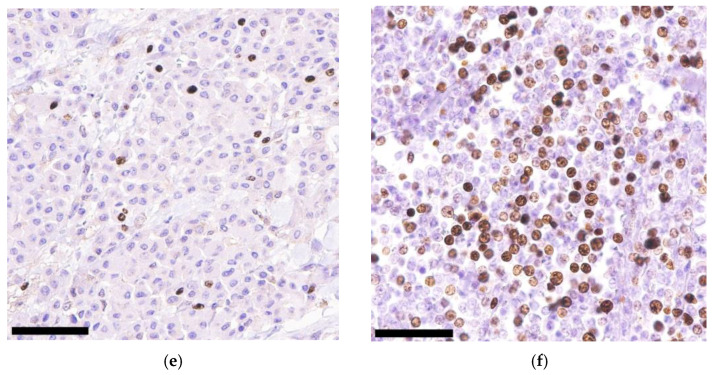
Macroscopic (**a**) and immunohistochemical ((**b**–**f**); bar = 60 µm) findings in different digital mast cell tumours from this study: (**a**) Longitudinal cut of a formalin-fixed amputated digit with a mass (asterisk) originating from the cutis and extending downwards; (**b**) KIT pattern I showing membrane-associated staining; (**c**) KIT pattern II with distinctive perinuclear staining; (**d**) KIT pattern III showing diffuse cytoplasmic staining; (**e**) Ki67 staining with less than 23 Ki67-positive cells per grid area; (**f**) Ki67 staining with more than 23 Ki67-positive cells per grid area.

The size of the tumours varied in diameter from 0.2 cm to 7.0 cm (median: 2.5 cm). Margins were clean in 29 cases, narrow in 6 cases, and in 33 cases, the margins were infiltrated by tumour cells. An example of the macroscopic appearance is given in Figure 1a.

The analysis based on the three-tier system of Patnaik and colleagues [8] resulted in the following: 15 × grade I (22.1%), 46 × grade II (67.6%) and 7 × grade III (10.3%). All cases were additionally graded according to the two-tier system of Kiupel and colleagues [10]. Some 59 cases (86.8%) were categorised as low-grade tumours, and 9 cases (13.2%) were high-grade tumours. All Patnaik grade I tumours were Kiupel low-grade, and all Patnaik grade III tumours were Kiupel high-grade. Of the 46 cases classified as Patnaik grade II, 44 cases (95.7%) were Kiupel low-grade tumours, and 2 cases (4.3%) were Kiupel high-grade tumours. In the Patnaik grading system, mitotic figures/HPF ranged from 0 to 4 (median: 1). For the Kiupel grading system and the statistical analysis, the mitotic figures/10 HPF were recorded. They ranged from 0 to 9 (median: 1).

Logistic regression models could not find any statistically significant relationship between the two grading systems with regard to gender, castration, tumour size and age. However, a high mitotic count was significantly associated with higher grading in both the Patnaik (*p* < 0.001) and the Kiupel (*p* < 0.001) grading systems.

### 3.3. Immunohistochemical KIT and Ki67 Expression

The distribution of the KIT staining pattern was as follows: 28 × staining pattern I (membrane-associated; 41.2%; Figure 1b), 9 × pattern II (focal to stippled cytoplasmic; 13.2%; Figure 1c), and 31 × pattern III (diffuse cytoplasmic; 45.6%; Figure 1d).

The Ki67 count was determined in 65 cases; in 3 cases, it was not possible to obtain any evaluable staining, despite repeating the process several times. The average count of Ki67-positive cells from five fields of 250 × 250 µm each ranged from 1.4 to 77.2 (median: 24.0). In this study, 34/65 tumours (52.3%) were above the threshold (more than 23 Ki67-positive cells per grid area), of which 6 (17.6%) were Patnaik grade I. Some 23 tumours (67.7%) were grade II (22 Kiupel low-grade, 1 Kiupel high-grade), and the remaining 5 (14.7) were grade III. Two examples of Ki67 staining are shown in Figure 1e,f.

**Figure 2:**
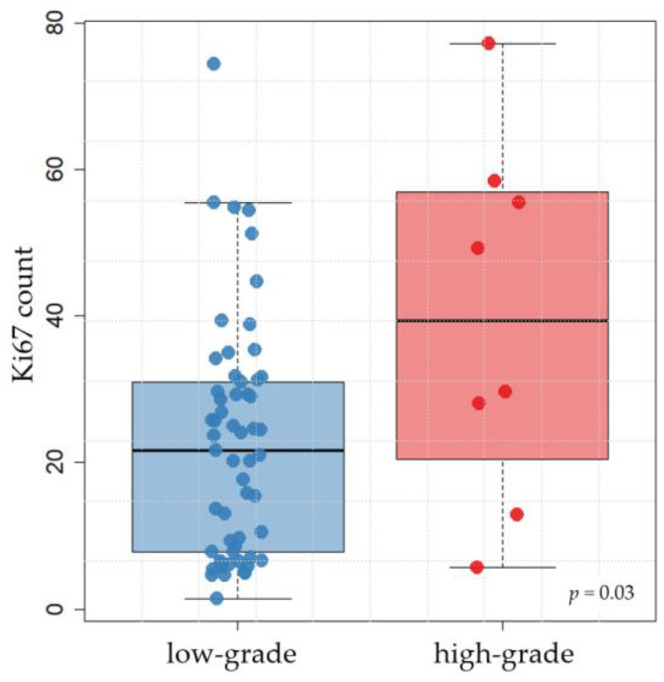
Distribution of Ki67-positive cells (average of five fields, each 250 × 250 µm) on the *y*-axis, within the Kiupel two-tier system on the *x*-axis. The association between the two parameters was statistically significant (*p* = 0.03).

Statistical analysis showed that the Ki67 count and KIT pattern had no statistically significant association with each other and, furthermore, both variables were not associated with Patnaik or Kiupel grading. The logistic regression compared a variety of factors (see Section 2.4) with the two grading systems. In both cases, the model selected only one factor: ITD in *c-kit* exon 11 (both *p* < 0.001). We omitted this significant factor and recalculated the model. We obtained a significant (*p* = 0.027) association between Patnaik grading and immunohistochemical KIT pattern. We also found a significant (*p* = 0.03) relationship between Kiupel grading and Ki67 count (Figure 2).

### 3.4. c-kit Mutation Status

In total, the *c-kit* mutation status of exons 8, 9, 11 and 14 was investigated in 68 dogs (*n* = 272 PCR approaches). In 233/272 cases (85.7%), amplification was successful and the corresponding product was sequenced. As far as the individual exons are concerned, the following number of cases could be sequenced: *n* = 53 for exon 8; *n* = 64 for exon 9; *n* = 63 for exon 11; and *n* = 53 for exon 14.

In the 53 tumours screened for *c-kit* exon 8 variants, neither ITDs nor any other mutations were found; all cases showed the sequence that corresponds to the wild type. The 64 cases examined for *c-kit* exon 9 mutations were all wild-type.

One case with a mutation was found in exon 14 with a G > A transversion (c.2020G > A), which causes an exchange of the amino acid glutamic acid to lysine. The tumour containing this mutation had no mutations in the other exons examined. The outcome data were not available.

**Figure 3:**
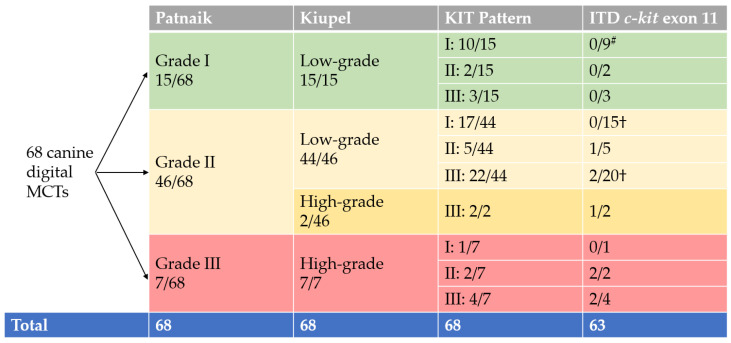
Summary of grading, immunohistochemistry (KIT) and screening for ITD in *c-kit* exon 11. Abbreviations and notes: MCTs, mast cell tumours; ^#^, PCR failed *n* = 1; †, PCR failed *n* = 2.

ITDs in *c-kit* exon 11 were found in 8/63 tumours (12.7%). In addition, in two cases, a single nucleotide polymorphism (SNP) was found in exon 11, which is also listed as such in the ENSEMBL gene database (SNP No.: rs853024368; https://www.ensembl.org/index.html, accessed on 23 January 2023). This is a silent polymorphism, as the amino acid tyrosine is retained despite the base exchange of C > T. Another SNP, consisting of a T > C base exchange, was found in 16/63 cases (25.4%) in the adjacent intron 11; this has already been described in the literature, and also has no effect on the constitution of the protein as it is located in the non-coding intron [43]. Graphical information on the results of grading, immunohistochemistry (KIT), and screening for ITD in *c-kit* exon 11 are summarised in Figure 3.

The statistical analysis examined whether the presence of mutations in *c-kit* was associated with the characteristics of the histological factors (grading according to Kiupel and Patnaik, as well as mitotic count) and the immunohistochemical results (KIT pattern and Ki67 count). The analysis was limited to *c-kit* exon 11, because no mutation was found in *c-kit* exons 8 and 9, and only one mutation was found in *c-kit* exon 14, which was considered insufficient for statistical significance. 

MCTs with an ITD in *c-kit* exon 11 were significantly (*p* = 0.002) associated with a higher Patnaik grading (grade II/III) compared with Patnaik grade I. Furthermore, the relationship between the presence of an ITD in *c-kit* exon 11 and the classification of the tumour as Kiupel high-grade was significant (*p* < 0.001). The majority of tumours without an ITD in *c-kit* exon 11 were classified as Kiupel low-grade (51/55; 92.7%). More than half (5/8; 62.5%) of the eight tumours with an ITD in *c-kit* exon 11 were Kiupel high-grade, but the remaining three (all Patnaik grade II) were Kiupel low-grade. ITDs in *c-kit* exon 11 were also associated with a generally increased mitotic count (*p* < 0.001) and a Ki67 count above the threshold of 23 positive cells per grid area (*p* = 0.006). Furthermore, data showed that tumours with an ITD in *c-kit* exon 11 tended to have an aberrant KIT pattern (*p* = 0.018). The *p*-values are summarised in Table 3. 

The most common purebred dogs in this study were French Bulldogs, Boxers, Retrievers and Pugs. Among the mixed breeds, the proportion of high-grade MCTs (1/13; 7.7%) and ITD in *c-kit* exon 11 (2/12; 16.7%) was low. Retrievers and Pugs all had low-grade tumours and no ITD in *c-kit* exon 11. One in six Boxers (16.7%) had a high-grade MCT, but none of them had an ITD in *c-kit* exon 11. Unexpectedly, French Bulldogs were more frequently affected by high-grade MCTs (4/7; 57.1%) and ITD in *c-kit* exon 11 (3/7; 42.8%) compared with mongrels. 

## 4. Discussion

The aim of this study was to examine canine digital MCTs histologically (with Patnaik and Kiupel grading) and immunohistochemically (with KIT pattern and Ki67 count) on a larger scale, and to provide an overview of *c-kit* mutation status. 

Digital MCTs are a subset of cutaneous MCTs. Studies that have investigated canine MCT in the past often did not differentiate between the exact anatomical location, and it can therefore not be excluded that digital MCTs were not part of these studies. When the tumour site was reported, it was often limited to the limb in general [4,7,22,29,32,44,45,46]. Thus, no conclusions could be drawn as to whether MCTs of the digit were part of the study material. A direct comparison of the data is therefore not possible. However, it was attempted at certain points of this study in order to be able to place the study results of digital MCTs in the overall context of cutaneous MCTs.

Breeds that seem to have a predisposition to developing cutaneous MCTs have already been identified [3,4,7,8], and some of them, such as French Bulldogs, Boxers, Retrievers and Pugs, were also represented in this study. A study by Śmiech et al. [4] found that French Bulldogs, Golden and Labrador Retrievers and Boxers were more likely to develop low-grade MCTs. In Golden and Labrador Retrievers, germline variants in the *DSCAM* gene have been reported, which could be responsible for the fact that these two breeds are frequently affected by the development of MCTs [47]. Pugs, too, have been described as being more prone to developing low-grade MCTs [45]. For Retrievers, Boxers and Pug Dogs, our results are in agreement. However, our findings differed from the aforementioned study by Śmiech et al. regarding French Bulldogs, as in our study of digital MCT, they were more likely to develop high-grade MCTs. Additionally, there was a higher occurrence of ITDs in *c-kit* exon 11 in this breed than in mongrels. This could indicate that French Bulldogs are a breed in which the location of MCTs at the digit plays a special role, as they had an increased proportion of poorly differentiated MCTs according to our results. Thus, *c-kit* mutation analysis, in addition to histological grading, could be particularly useful in identifying poorly differentiated MCTs in French Bulldogs. It should be mentioned that the majority of tumours investigated in this study were from German dogs. French Bulldogs are a popular dog breed in Germany, which has been confirmed by other studies analysing data from a German animal registration portal [3,35]. The number of French Bulldogs in our study population thus reflects the breed distribution in Germany. The proportion of certain dog breeds, such as French Bulldogs, in the total animal population may therefore vary between different regions and countries.

The majority (67.6%) of the digital MCTs examined in this study were Patnaik grade II. This is consistent with what was observed by colleagues who graded cutaneous MCTs from different sites and also found Patnaik grade II to be the most common grade, ranging from 43% to 75% [8,46,48,49]. Most (86.8%) of the digital MCTs corresponded to Kiupel low-grade. All Patnaik grade I tumours were Kiupel low-grade, and all Patnaik grade III tumours were Kiupel high-grade, while for Patnaik grade II, the majority (95.7%) could be classified as Kiupel low-grade tumours. A review by Avallone et al. [12] described similar ratios of the two grading systems when applied to cutaneous MCTs in general. We therefore conclude that the ratios found in the histological grading of digital MCTs, as a subset of cutaneous MCTs, are comparable to the data shown in the literature for cutaneous MCTs.

In the prognostic assessment of canine MCT, the two immunohistochemical markers Ki67 and KIT are frequently used. They were also recommended in a recent consensus article [50], and were used in our study on digital MCT. A meta-analysis by Freytag et al. [13] confirmed the prognostic value of Ki67 and KIT, and confirmed the legitimacy of these immunohistochemical markers for use in routine diagnostics. In their meta-analysis, the colleagues also identified the protein BAX, which is involved in apoptosis, as an immunohistochemical marker that could be used in the future in MCTs, as it has promising prognostic value [13]. Thamm et al. [51] investigated phosphorylated KIT (pKIT) and found that its immunohistochemical expression is a predictor of the biological behaviour of cutaneous MCTs. However, the authors also emphasised that further studies are needed before a recommendation can be made for the use of pKIT in routine diagnostics. Proliferating cell nuclear antigen (PCNA) and the argyrophilic nucleolar organizing region (AgNOR) are proliferation markers that have been investigated by Webster et al. [30], in addition to Ki67, for their independent prognostic significance. The routine use of AgNOR and Ki67 was recommended to accompany histological grading, *c-kit* mutation analysis and KIT immunohistochemistry in order to assess the progression of canine MCT [30].

It can be found in the literature on cutaneous MCTs that an aberrant immunohistochemical staining pattern of the KIT receptor [16], an increased Ki67 count of more than 23 Ki67-positive cells per grid area [30], and a high mitotic count [52] correlate with a higher histological grade. In our study, although most of the MCTs were prognostically favourable according to the grading, more than half of the tumours (52.3%) had a Ki67 count of more than 23 positive cells per grid area, a limit defined by Webster et al. [30] for cutaneous MCTs. In addition, well over half of the cases (58.8%) had an aberrant KIT pattern. Even though the statistical analysis did not show a significant relationship between KIT pattern and Ki67 count, logistic regression models demonstrated a statistical significance between Patnaik grading and KIT pattern (*p* = 0.027), and between Kiupel grading and Ki67 count (*p* = 0.03). Furthermore, there was a significant association (*p* < 0.001) between a high mitotic count and a higher grading (in both systems). The large proportion of tumours in our study with aberrant KIT patterns and Ki67 counts above the threshold were an unexpected finding, and suggest that despite the fact that many of the tumours were histological low-grade tumours, digital MCT might be prognostically unfavourable. This retrospective study could not provide a meaningful outcome analysis, and future prospective studies will be necessary to evaluate the prognostic significance of KIT and Ki67 immunohistochemistry in digital MCT.

Some 8 of the 63 cases (12.7%) were found to have an ITD in *c-kit* exon 11. The additional identification of two different types of SNP in exon and intron 11 shows that, concerning its results, the methodology was robust. ITDs in *c-kit* exon 11 were found in tumours that generally had a higher Patnaik (*p* = 0.002) and Kiupel (*p* < 0.001) grading, a higher mitotic count (*p* < 0.001), an increased number of Ki67-positive cells *(p* = 0.006), and an aberrant KIT pattern (*p* = 0.018). It should be mentioned here, however, that the connection between *c-kit* mutations and aberrant expression of KIT is not as clear as it appears, as they seem to be independent processes [53]. The present data confirm that ITDs in *c-kit* exon 11 may be a tool for identifying histologically aggressive canine digital MCT.

No mutations were found in *c-kit* exon 8. Mutations located in this exon, mainly ITDs, have been described in cutaneous MCT as being associated with a more favourable prognosis [26]. In a recent study by Chen et al. [54] on 216 subcutaneous MCTs, 10.6% of cases were identified as carrying a *c-kit* exon 8 ITD, and only 5.6% of cases were found to have an ITD in exon 11, a ratio that is the inverse of what has been described for cutaneous forms. The authors concluded that subcutaneous MCTs tend to be characterised by a less aggressive biological behaviour than the cutaneous MCTs. Overall, mutations in *c-kit* exon 8 in digital MCT, being associated with a potentially better prognosis, seem to play a minor role, as they could not be found in our study.

No mutations were found in *c-kit* exon 9 of digital MCTs. Since mutations in this exon are discussed as being partly responsible for the development of resistance to the RTKI masitinib [27], it was in our interest to quantify them in digital MCTs. They are rather rare in cutaneous MCTs [22,24], and were absent in our study population of digital MCTs.

A single point mutation was found in *c-kit* exon 14. Mutations in this exon are also rare in cutaneous MCT; however, there is one case in which a deletion in *c-kit* exon 11 was found in a lymph node metastasis of a canine MCT, in addition to a second mutation in *c-kit* exon 14 [25]. The colleagues demonstrated that this second mutation in exon 14 caused resistance to the RTKI imatinib in vitro. This gave us the incentive to include exon 14 in our investigations. However, our case with a point mutation in exon 14 showed wild-type sequences in the other exons, and the further clinical course was unknown.

Concerning the aim of this study, it was possible to extensively examine the digital MCTs histologically and immunohistochemically, and to detect their *c-kit* mutation status. Unexpectedly, many digital MCTs had an aberrant immunohistochemical KIT pattern and were highly proliferative, despite a large proportion of low-grade tumours. In certain breeds, such as French Bulldogs, it may be particularly useful to consider the anatomical location of the MCT in a differentiated way, as the digits of this breed may more often have poorly differentiated MCTs. What remains is the lack of outcome analyses that could have further investigated the prognostic significance of grading, immunohistochemical KIT patterns, Ki67 activity and *c-kit* status. The limitations of this study arose from its retrospective nature. Under these circumstances, it was also not possible to differentiate whether the animals were affected by solitary or multiple MCTs, and if metastases occurred. A possible approach for future prospective studies would be to compare digital MCTs with a group of distinctly non-digital MCTs, in order to make reliable statements about the extent to which digital MCTs differ from those at other sites. This could further contribute to our understanding of this tumour site.

## 5. Conclusions

In summary, only a few of the digital MCTs were histologically poorly differentiated (Patnaik grade III or Kiupel high-grade). Nevertheless, it is remarkable that a high proportion of tumours had aberrant KIT patterns II and III, and many digital MCTs also had increased proliferation activity, as detected by Ki67. In addition to histological grading, testing for ITD in *c-kit* exon 11 in digital MCTs might be useful for identifying aggressive tumours, especially in French Bulldogs, as a statistically significant association with higher grading, an aberrant KIT pattern and an increased Ki67 count could be found.

## Figures and Tables

**Table 1:** Primary antibodies and pre-treatment used for immunohistochemistry.

Antigen	Species	Supplier	Dilution	Pre-Treatment
KIT/CD117	rabbit	Dako ^1^ (#A4502)	1:150	EDTA buffer
Ki67	mouse	Dako ^1^ (#M7240)	1:200	EDTA buffer

^1^ Dako Denmark A/S, Glostrup, Denmark.

**Table 2:** Primer sequences used for the analysis of *c-kit* exons 8, 9, 11 and 14.

*c-kit* Exon	Primer	Sequence
8	Forward Reverse	5′-GGT GAG GTG TTC CAG CAG TC-3′ 5′-CCT TCC CTC GTG CAC ATT A-3′
9	Forward Reverse	5′-ACT CGT CTC TGT CAC CGT CTG GAA-3′ 5′-ATG GCA GGC AGA GCC TAA ACA TCC-3′
11	Forward Reverse	5′-CCC ATG TAT GAA GTA CAG TGG AAG-3′ 5′-GTT CCC TAA AGT CAT TGT TAC ACG-3′
14	Forward Reverse	5′-AGC CTG CTA AGT ATT TGC CTT ATC AAT-3′ 5′-TGG CAC CTC GAA GTA CCT CTG T-3′

**Table 3:** Significant relationships between internal tandem duplication (ITD) in *c-kit* exon 11 and histological/immunohistochemical parameters.

Parameter	ITD *c-kit* Exon 11
Patnaik grading	*p* = 0.002
Kiupel grading	*p* < 0.001
Mitotic count	*p* < 0.001
Ki67 count	*p* = 0.006
KIT pattern	*p* = 0.018

## Data Availability

Data are available upon request due to privacy/ethical restrictions.

## Supplementary Materials

The following supporting information can be downloaded at: https://www.mdpi.com/article/10.3390/ani13101694/s1, Table S1: Mutational analysis, histological and immunohistochemical findings and case description of dogs with digital mast cell tumours (*n* = 68).
